# Discovery and Synthesis of Hydroxy-l-Proline Blockers of the Neutral Amino Acid Transporters SLC1A4 (ASCT1) and SLC1A5 (ASCT2)

**DOI:** 10.3390/molecules29102330

**Published:** 2024-05-15

**Authors:** Brent R. Lyda, Gregory P. Leary, Jill Farnsworth, Benjamin Seaver, Derek Silvius, Michael P. Kavanaugh, C. Sean Esslinger, Nicholas R. Natale

**Affiliations:** 1 Division of Biological Sciences, University of Montana, 32 Campus Dr., Missoula, MT 59812, USA; 2 Department of Biomedical and Pharmaceutical Sciences, University of Montana, 32 Campus Dr., Missoula, MT 59812, USAbenjamin.seaver@mso.umt.edu (B.S.); derek@mclaughlinresearch.org (D.S.);; 3 Medicinal Chemistry Graduate Program, University of Montana, 32 Campus Dr., Missoula, MT 59812, USA

**Keywords:** solute carrier superfamily (SLC), alkoxy hydroxy-pyrrolidine carboxylic acids (AHPCs), computational homology model, SLC1A4 (ASCT1), SLC1A5 (ASCT2), electrophysiology

## Abstract

As a conformationally restricted amino acid, hydroxy-l-proline is a versatile scaffold for the synthesis of diverse multi-functionalized pyrrolidines for probing the ligand binding sites of biological targets. With the goal to develop new inhibitors of the widely expressed amino acid transporters SLC1A4 and SLC1A5 (also known as ASCT1 and ASCT2), we synthesized and functionally screened synthetic hydroxy-l-proline derivatives using electrophysiological and radiolabeled uptake methods against amino acid transporters from the SLC1, SLC7, and SLC38 solute carrier families. We have discovered a novel class of alkoxy hydroxy-pyrrolidine carboxylic acids (AHPCs) that act as selective high-affinity inhibitors of the SLC1 family neutral amino acid transporters SLC1A4 and SLC1A5. AHPCs were computationally docked into a homology model and assessed with respect to predicted molecular orientation and functional activity. The series of hydroxyproline analogs identified here represent promising new agents to pharmacologically modulate SLC1A4 and SLC1A5 amino acid exchangers which are implicated in numerous pathophysiological processes such as cancer and neurological diseases.

## 1. Introduction

The amino-acid transporters SLC1A4 (ASCT1) and SLC1A5 (ASCT2) are sodium dependent transmembrane proteins that mediate the exchange of neutral, largely polar l-amino acids [1,2,3,4]. Both transporters belong within a superfamily of organelle- and plasma membrane-spanning proteins known as solute carriers (SLCs) which transport solutes independently of ATP hydrolysis, thereby distinguishing them from the ABC transporter superfamily. SLCs encompass over 400 members across 65 distinct gene families responsible for transporting an array of biomolecules and essential nutrients, including amino acids, neurotransmitters, sugars, nucleotides, fatty acids, inorganic ions, drugs, and complex organic molecules [5,6]. Several SLC families are targets of drugs in clinical use (e.g., SLC6 and/or SLC12 families). Although numerous remaining SLCs are implicated in human disease, the development of therapeutic agents targeting these specific transporters has been hindered by incomplete substrate and structural characterization, as well as deficits in understanding their respective physiologic functions [7], including those of SLC1A4 and SLC1A5. Nevertheless, both novel and traditional small-molecule approaches that modulate SLC function offer great promise for advancing medicine and diagnostics.

First characterized and denoted as ASC (Ala, Ser, Cys) based upon their representative substrates alanine, serine, and cysteine [8], SLC1A4 and SLC1A5 exhibit generally similar substrate profiles but differ notably with respect to glutamine, a substrate of only SLC1A5 [1,3,9]. While plasma membrane transport of amino acids mediated by these proteins is Na^+^-dependent, it occurs by an obligate substrate exchange mechanism [10]. In contrast, the other SLC1 paralogs (EAATs 1–5) are acidic amino acid transporters (glutamate/aspartate) that pairs substrate transport to the electrogenic flux of three Na^+^ ions, one proton, and the countertransport of a potassium ion [11,12]. Despite these distinctions, all members of the SLC1 family maintain a chloride conductance thermodynamically uncoupled to substrate flux. This anion current is the sole measurable current among the neutral amino acid exchangers of the SLC1 family [10,13].

Noticeable gaps still exist in our understanding of how SLC1A4 and SLC1A5 participate in human disease. SLC1A5 is broadly expressed in tissues and maintains homeostasis of the centrally important amino acid glutamine [3,4]. SLC1A5 has long been a focus of investigation in cancer pathophysiology, where it is noted to be upregulated in numerous cancer cell lines by metabolic transcriptional regulators such as c-MYC and the kinase mTOR, which governs cancer cell proliferation, growth, and survival [14,15,16,17,18,19]. Blocking SLC1A5 function through CRISPR knockout or mRNA silencing has been shown to suppress tumor growth and metastasis, emphasizing the need to develop SLC1A5specific inhibitors for evaluation as antiproliferative/anticancer agents [20,21,22,23,24,25].

While SLC1A4 is widely expressed in mammalian tissues, its highest expression is observed in the brain [1,2]. Underscoring the significance of SLC1A4 within the nervous system, mutations in the corresponding human gene have been linked to neurological disorders, including cognitive and developmental impairment [26,27,28,29]. Besides the exchange of l-amino acids, SLC1A4 expressed within astrocytes is known to mediate transport of the neurotransmitter d-serine [30,31,32], a primary co-agonist of the NMDA receptor. NMDA receptors are fundamental in excitatory neurotransmission and neuronal plasticity across the brain. Targeting the transport mechanisms that control reuptake of d-serine presents a novel therapeutic approach for neuropsychiatric disorders characterized by hyper- or hypo-functioning NMDA receptors, such as in schizophrenia, PTSD, or depression [33,34]. Moreover, inhibition of SLC1A4 could prove beneficial in conditions involving traumatic brain injury and Alzheimer’s disease, wherein studies performed with animal models have demonstrated that d-serine efflux from inflammatory astrocytes can exacerbate NMDA-mediated excitotoxicity [35,36,37,38,39].

Pharmacologic tools are critically needed to assess the therapeutic benefit of modulating SLC1A4 and SLC1A5 transporter function within human-associated diseases. Thus far, there has been limited progress towards developing selective and/or potent inhibitors of SLC1A4 or SLC1A5 for in vivo or preclinical evaluation. Non-transportable SLC1A5 competitive inhibitors based on l-*γ*-glutamyl-*p*-nitroanilide, benzyl-serine or benzyl-cysteine have been reported [40,41,42]. Although several of these substrate derived analogs initially demonstrated modest selectively towards SLC1A5, they have little utility as pharmacologic tools in translational research due to low affinity, poor pharmacokinetic properties, or inhibition among SLC paralogs. Additionally, ligands based on l-proline have shown improved affinities for inhibiting SLC1A5 to within the lower micromolar range, where again activity across other SLC paralogs was not evaluated [43,44]. 

We sought to generate a new class of SLC1A4 and SLC1A5 inhibitors with high affinity and selectivity through employing conformational restriction provided by functionalized pyrrolidines. The neutral amino acid l-proline does not elicit detectable chloride currents at concentrations up to 1 mM in oocytes expressing SLC1A4 [1]. Nevertheless, application of extracellular 300 µM l-proline is capable of inducing heteroexchange release of intracellular radiolabeled ASCT substrates, indicating that it is a transportable substrate [10]. The 4*R*- substituted alcohol of *trans*-4-hydroxy-l-proline **2** contributes to an increase in affinity for SLC1A4 by approximately 20-fold over l-proline [45]. From a synthetic and computational perspective, pyrrolidines are particularly useful for designing amino acid analogs to introduce functional group diversity that occupy unique chemical space and offer limited rotational freedom, such as analogs of neurotransmitters [46]. We now report the synthesis, computational docking, and functional characterization of a novel series of hydroxyproline analogs. Using electrophysiological approaches, we observe that a series of hydroxyprolines, alkoxy hydroxy-pyrrolidine carboxylic acids (AHPCs), demonstrate inhibitory activity, targeting SLC1A4 and SLC1A5 transporters with affinities in the low micromolar and nanomolar range.

## 2. Results

### 2.1. Hydroxy Proline Scaffold 

**Figure 1:**
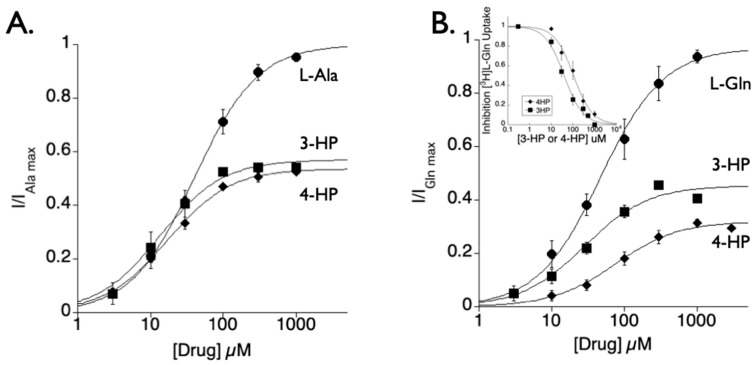
The scaffolds 3-HP and 4-HP dose-dependently activate SLC1A4 and mSLC1A5 transporter currents. (**A**) SLC1A4 dose response curves for substrate activated anion currents using two-electrode voltage clamp of *Xenopus lavies* oocytes (−30 mV). Currents are normalized to the maximum current estimated for alanine from the Michaelis-Menten equation fit (In µM; Ala K_m_ = 40.3 ± 1.8; 3-HP K_m_ = 13.5 ± 1.8; 4-HP K_m_ = 17.1 ± 0.8). (**B**) SLC1A5 dose response curve for substrate activated anion currents using two-electrode voltage clamp (−30 mV). Currents are normalized to the maximum current estimated for glutamine from the Michaelis-Menten equation fit (In µM; Gln K_m_ = 47.4 ± 4.6; 3-HP K_m_ = 28.6 ± 6.4; 4-HP K_m_ = 76.3 ± 11.0). Extracellular solutions contained 50 mM NaSCN*. Inset B:* 10 µM*[^3^H]l-Gln uptake in SLC1A5 is blocked dose-dependently by both 3-HP (IC_50_ = 39 µM) and 4-HP (IC_50_ = 108 µM).

*Trans*-3-hydroxy-l-proline (3-HP) and *trans*-4-hydroxy-l-proline (4-HP) were evaluated as substrates of SLC1A4 and SLC1A5 with two-electrode voltage clamp (TEVC) measuring substrate induced SCN^−^ anion currents, a technique found to be reliable for screening and characterizing transport kinetics of the SLC1A4 and SLC1A5 transporters [6,9]. Application of 3-HP or 4-HP to oocytes expressing SLC1A4 or SLC1A5 revealed anion currents that were concentration dependent and saturable. These dose response curves were normalized to the maximal response induced by the principal substrate, l-Ala or l-Gln, respectively (Figure 1). The dose responses were fit by the Michaelis-Menten equation (Figure 1A_ SLC1A4: 3-HP K_m_ = 13.5 ± 1.8 μM; 4-HP K_m_ = 17.1 ± 0.8 μM; l-Ala K_m_ 40.0 ± 1.8 μM or Figure 1B_ SLC1A5: 3-HP K_m_ = 28.6 ± 6.4 μM; 4-HP K_m_ = 76.3 ± 11.0 μM, l-Gln K_m_ = 47.4 ±4.6 μM). For both molecules, there was a higher apparent affinity for SLC1A4 compared with SLC1A5. Radiolabeled uptake of [H^3^]l-Gln was dose dependently blocked by 3-HP and 4-HP, confirming the affinity for SLC1A5. Additionally, 4-HP applied at 1 mM was used to assess activity at the glutamate transporters SLC1A3 or SLC1A1. No TEVC current response or block was observed, which is expected considering both transporters are known to preferentially transport carboxylic acid side chain l- and d- amino acids. However, 4-HP did induce currents when applied to the neutral amino acid transporter SLC38A2 at millimolar concentrations (SLC38A2_4-HP, K_m_ = 3.1 mM).

### 2.2. Design of SLC1A4 and SLC1A5 Inhibitors

#### Analogs of Hydroxy-l-Proline: Design

**Figure 2:**
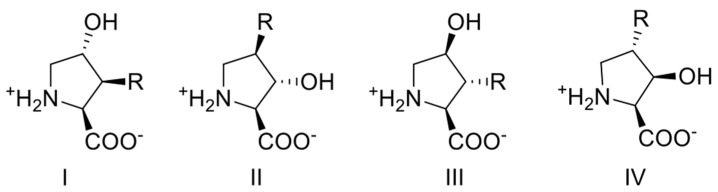
Multi-functionalized hydroxy-l-proline analog targets to be synthesized and assessed for inhibitory activity at SLC1A4 and SLC1A5. R = any group.

As a scaffold, Hydroxy-l-prolines offer appealing targets owing to both their degree of functionalization and conformational restriction afforded through the pyrrolidine heterocycle. Hydroxyprolines with an array of various stereo- and regiochemical configurations were designed to survey binding orientation/mode and scan for polar interactions within the orthosteric binding site of SLC1A4 and SLC1A5 (Figure 2). From these concept designs, a series of diverse multi-functionalized pyrrolidines were synthesized from protected *cis* or *trans*-3,4-epoxy-l-prolines including regioisomers of methyl, phenyl or phenol ether substituted hydroxy-l-prolines, compounds **10**[**a–d**], **12a** and **12b**; benzyl or alkyl ether substituted AHPCs, compounds **14**[**a–f**], **17**[**a–c**]; and finally, two 4,4-gem substituted 4-hydroxyproline derived from the intermediate *N*-protected 4-keto-proline, *cis*-4-methyl-*trans*-4-hydroxy-l-proline **26**, and *cis*-4-hydroxy-*trans*-4-methyl-l-proline **27**.

Stereochemistry and regiochemistry of 3,4-substituted pyrrolidine products were established from the fully protected (*N*-Cbz and benzyl ester) 3,4-epoxy-prolines from which they were derived. The *cis* and *trans* epoxide isomers are easily isolatable allowing stereochemistry to be determined by 2D-NOE. Our stereo-chemical assignments of both isomers are consistent with that previously reported [47]. Stereochemistry of geminal substituted 4-methyl-4-hydroxy-l-proline was deduced directly as the final product via 2D-NOE. Regiochemical assignments of final substituted hydroxyprolines were inferred by a combination of 2D gradient correlation spectroscopy (gCOSY) for all final products in addition to 2D-NOE (for **14a**, **14b** and **17a** only). Supplemental Figure S1 illustrates how the proton assignments and regiochemistry were made from a gCOSY experiment of example AHPC, *trans*-3-hydroxy-*cis*-4-isopropoxy-l-proline **14b**. Refer to the experimental procedures section for full experimental details, procedures, and synthetic schemes. 

### 2.3. Synthesis of Hydroxyproline Analogs

#### 2.3.1. *N*-Cbz-(*Cis*- or *Trans*)-3,4-Epoxy-l-Proline Benzyl Ester

**Scheme 1:**
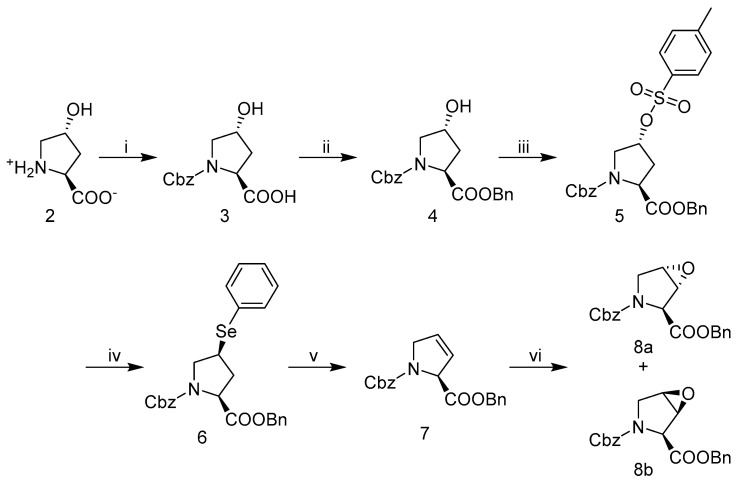
Synthesis of *N*-Cbz-*trans*- and *cis*-3,4-epoxy-proline-benzyl ester **8a** and **8b**. (i) benzylchloroformate, 1:1 MeOH:H_2_O, NaOH, rt; (ii) benzylbromide, Na_2_CO_3_, cat NaI in DMF, rt; (iii) p-toluenesulfonyl-Cl, pyridine, 0 °C; (iv) diphenyl diselenide, NaBH_4_ in tBuOH, reflux; (v) CH_2_Cl_2_, H_2_O_2_ in H_2_O, 0 °C-RT; (vi) mCPBA, CH_2_Cl_2_, reflux.

To ultimately arrive at hydroxyprolines I-IV (Figure 2), we adapted a route to convert *trans*-4-hydroxy-l-proline **2** to the protected *trans*- and *cis*-3,4-epoxides (**8a** and **8b**) depicted in Scheme 1 with a slight modification to that previously reported [47]. Briefly, the amine group of *trans*-4-hydroxy-l-proline **2** was protected using benzylchloroformate. The carboxylic acid of *N*-Cbz-*trans*-4-hydroxy-l-proline **4** was then benzyl protected with benzylbromide, Na_2_CO_3_, NaI, in DMF. Intermediate **5** (*N*-Cbz-4-tosyl-l-proline benzyl ester) was obtained through treatment of the protected hydroxy-proline **4** with *p*-toluenesulfonyl chloride in pyridine at 4 °C. Substitution of tosylate intermediate **5** was accomplished under refluxing *tert*-butyl alcohol with reduced phenyl selenide providing the 4-phenylseleno-proline intermediate **6**. Post isolation, the phenylselenyl pyrrolidine intermediate was treated with H_2_O_2_ to obtain the olefin, protected 3,4-dehydro-l-proline **7**. Intermediate **7** was then converted to the protected *trans*- and *cis*-3,4-epoxy-prolines (**8a** and **8b**; Scheme 1) using *meta*-chloroperoxybenzoic acid (mCPBA) in dichloromethane, with reflux. Both fully protected *trans*- and *cis*-epoxides (**8a** or **8b**) were efficiently isolated in modest yields, ~40% up to this point within the synthetic scheme and utilized in the following epoxide ring opening additions under nucleophilic and/or Lewis acid initiated conditions.

#### 2.3.2. Alternative Synthesis of *N*-Cbz-3,4-Dehydro-l-Proline-Benzyl Ester **7** or *N*-Cbz-3,4-Dehydro-l-Proline **7a**

**Scheme 2:**
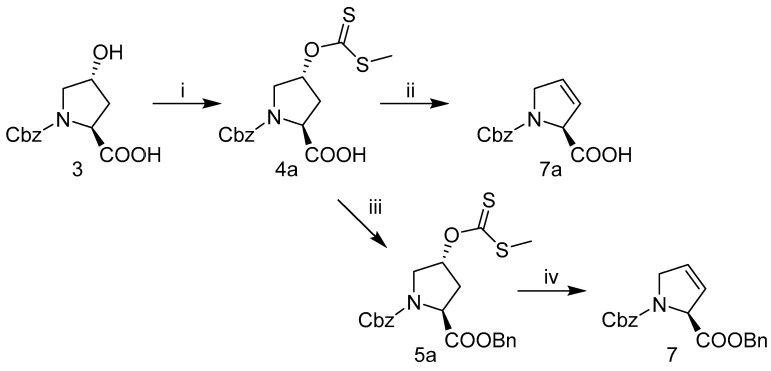
Alternative synthesis of 3,4-dehydroprolines **7a** and **7**. (i) NaH, THF, CS_2_, iodomethane, 22 °C; (ii) H_2_O, NaHCO_3_, microwave 100 °C; (iii) benzylbromide, Na_2_CO_3_, NaI, rt; (iv). H_2_O, Na_2_CO_3_, microwave 100 °C.

Alternative routes to produce 3,4-dehydro-l-prolines were explored through pyrolytic elimination of methyl xanthate ester pyrrolidine intermediates **4a** or **5a**, Scheme 2. Briefly, *N*-Cbz-*trans*-4-hydroxy-l-proline **3** was used in an esterification with CS_2_ and iodomethane using NaH in THF to produce *N*-Cbz-*trans*-4-methylxanthate-l-proline **4a**. Both 4-methyl xanthate pyrrolidines **4a** and benzyl protected pyrrolidine **5a** were successfully converted to the olefin under microwave heating in an open / unsealed flask, with sodium bicarbonate/or carbonate and water to provide the 3,4-dehydroprolines **7** and **7a** with acceptable yields. Further optimization of this pyrolytic elimination route would be beneficial to improving yields and to identify alternative conditions that pose a reduced risk of racemization than when using a carbonate base. Although pyrrolidine olefin intermediates **7** and **7a** are furnished under Scheme 2 with fewer synthetic steps, Scheme 1 proved to be amenable for scale-up and was therefore used to generate hydroxyproline analogs (Figure 2). 

#### 2.3.3. Methyl-, Phenyl-, or Phenol Ether Hydroxyprolines 

**Scheme 3:**
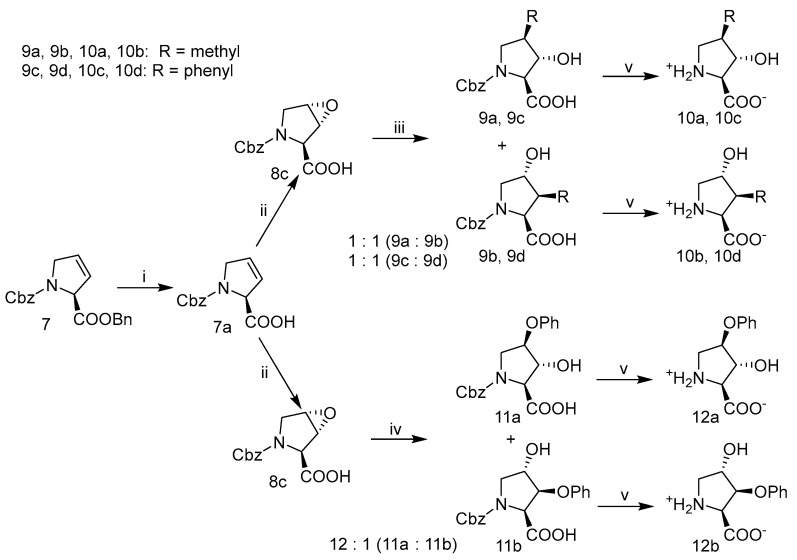
Synthesis of alkyl, aryl, or phenol ether hydroxyprolines: (i) KOH, THF:H_2_O, 0 °C; (ii) mCPBA, CH_2_Cl_2_; (iii) 2 eq. R_2_Cu(CN)Li_2_, Et_2_O, −40 °C under Ar_g_; (iv) sodium phenoxide in THF, 0 °C-rt; (v) 10% Pd/C (10% weight. eq), 40 psi H_2_, MeOH, H_2_O, and NH_4_OAc. 9a, 9b, 10a, 10b; R = methyl. 9c, 9d, 10c, 10d; R = phenyl.

Alkyl, aryl and phenoxy substituted hydroxyprolines were generated via nucleophilic addition. As precaution to avoid or minimize racemization of the α- chiral center during the addition step, free acid pyrrolidine epoxide intermediated **8c** (Scheme 3) was used. Treatment of the free acid olefin **7a** with mCPBA in DCM under reflux produced the *trans*-epoxide **8c** in excellent yields with no detectable *cis*-epoxide. Alternatively, epoxidation using dimethyldioxirane, generated in-situ*,* or peracetic acid added in excess also afforded epoxide **8c**, albeit, in low yields. The *N*-protected substituted hydroxyproline intermediates **9a** and **9b** or **9c** and **9d** were obtained from the free acid epoxide pyrrolidine by transformation of the organolithium reagent to a higher order cyano-cuprate complexation between two equivalents of organolithium reagent and CuCN (Scheme 3; iii) [48,49]. The resulting intermediates, three or four substituted *N*-protected hydroxyprolines, were recovered in crude-yields of 90–95% and the regioisomers were isolated via reverse phase HPLC in ratios of nearly 1:1.

Similarly, the *N*-protected phenoxy intermediates, **11a** and **11b**, were obtained from the free acid *trans*-epoxide **8c** under basic conditions via addition of sodium phenoxide with regioisomeric ratios of 12:1, 3-hydroxy: 4-hydroxy, respectively, with modest yields of ~80%. Hydrogenolysis of the *N*-benzylcarbamate (Cbz) intermediates **9a**–**d**, **11a**, and **11b** in AcOH and subsequent separation of the regioisomers via C18-reverse phase HPLC afforded the final hydroxyproline products **10a**–**d**, **12a**, and **12b**.

#### 2.3.4. Alkoxy Hydroxy-Pyrrolidine Carboxylic Acids (AHPCs)

**Scheme 4:**
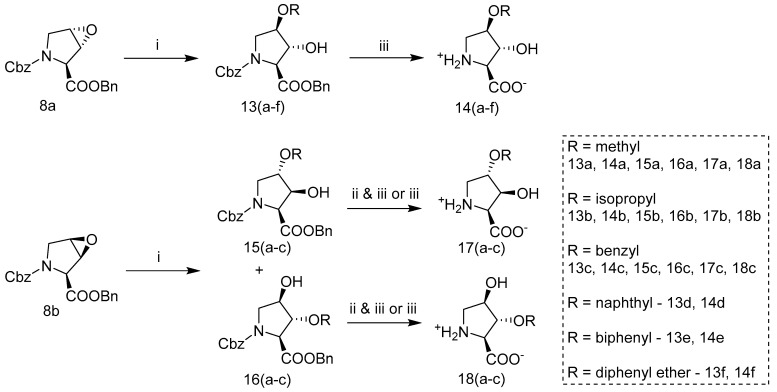
Synthesis of ether substituted AHPCs. (i) BF_3_·Et_2_O, CH_2_Cl_2_, desired primary alcohol (HOMeR), 22 °C; (ii) KOH, THF, H_2_O; (iii) 10% Pd/C (10% weight. eq.), H_2_, MeOH, H_2_O, ^+^NH_4_^−^OAc (0.5 eq). 13–18 (a) R = methyl; 13–18 (b) R = isopropyl; 13–18 (c) R = benzyl; 13–14 (d) R = naphthyl; 13–14 (e) R = biphenyl; 13–14 (f) R = diphenyl ether.

Ether substituted hydroxyprolines were synthesized and explored as potential inhibitors of the SLC1A4/5 transporters. Assembly of this series was accomplished by BF_3_•Et_2_O initiated nucleophilic ring opening of epoxide **8a** or **8b** with a primary alcohol and subsequent Pd-catalyzed hydrogenolysis to produce *trans*-hydroxy **14(a**–**f)** or, alternatively, to produce *cis*-hydroxy products **17** and **18** following ester hydrolysis, separation of the regioisomers, and finally hydrogenolysis (Scheme 4) (as specified in supplementary). Nucleophilic addition of alcohols catalyzed with BF_3_•Et_2_O provided superior yields compared to addition attempts initiated with *p*-toluenesulfonic acid that produced byproducts, including fortuitous addition of the tosylate ester later utilized to produce (2S,3R,4S)-3-hydroxy-4-phenoxypyrrolidin-2-carboxylate **12c,** see Supplemental Figure S4. Ring opening of *trans*-epoxide **8a** under BF_3_•Et_2_O initiated alcohol nucleophilic addition produced one regioisomer exclusively, the *N*-Cbz-*trans*-3-hydroxy-*cis*-4-ether-l-proline benzyl ester **13**. Whereas the ring opening of the *cis*-epoxide **8b** produced both regioisomer intermediates **15** and **16** in ratios, 8:1 (as approximated by ^1^H NMR) (Figure S5), greatly favoring the 3-positioned alcohol intermediates **15(a**–**c)**, consistent with azide or HCl ring opening of these epoxides [47,50]. Separation of these regioisomers was carried out by reverse phase HPLC following hydrolysis of the benzyl ester with KOH or just after hydrogenolysis. Of note, though not fully explored, we observed that *cis*-3-alkyl-ether-*trans*-4-hydroxy-l-proline isomers **14** may also be obtained from free acid *trans*-3,4-epoxy-proline **8c** (Scheme 3) via BF_3_•Et_2_O initiated addition under similar conditions. Benzyl carbamate deprotection of ether substituted intermediates **13, 15**, or **16** was again accomplished via palladium catalyzed hydrogenolysis under H_2 (g)_ affording final products **14**, **17**, or **18**. Importantly, we found that selective hydrogenolysis could be achieved via addition of ammonium acetate (NH_4_OAc) as an amine-based inhibitor of debenzylation during palladium catalyzed hydrogenolysis [51,52]. 

### 2.4. Pharmacology, Functional Screening of Substituted Hydroxyprolines

#### 2.4.1. Hydroxyproline Analogs Inhibit SLC1A4 and SLC1A5

**Figure 3:**
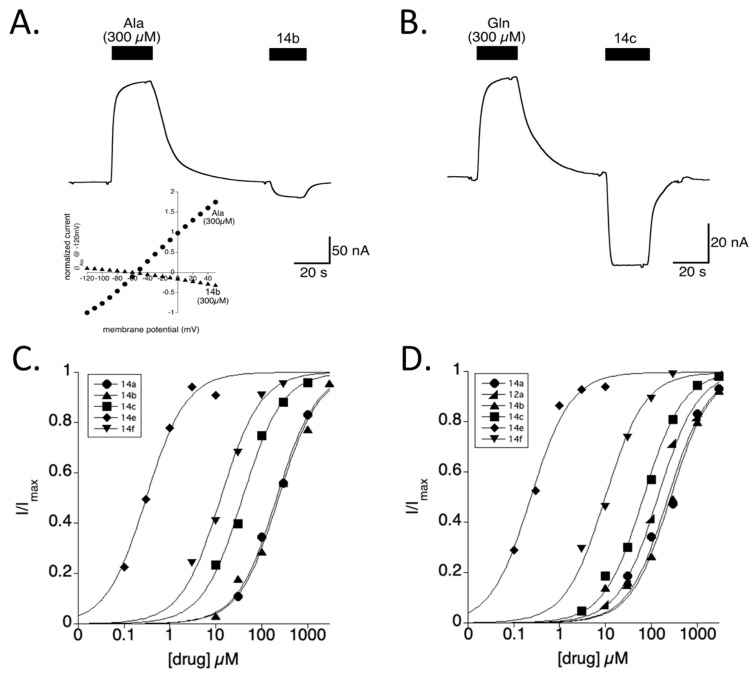
AHPCs block a leak current in SLC1A4 and SLC1A5. (**A**,**B**) Representative traces showing the anion current gated by substrates (l-alanine and l-glutamine) or blocked by the AHPCs **14b** or **14c** (at 300 µM) for SLC1A4 and SLC1A5, respectively. Inset (**A**): The current and voltage relationship for SLC1A4 currents activated by Ala or blocked by **14b** are determined by subtracting voltage jumps in buffer. The leak currents observed with SLC1A4 and SLC1A5 possess a saturable block (**C**,**D**), revealed through increasing concentrations of **14e** (down triangle), **14f** (diamond), **14b** (square), **14c** (up triangle), **12a** (wedge), and **14a** (circles). The 50% block is considered the K_i_ of binding for non-transportable ligands.

Inhibitors of SLC1A5 have been shown to block an anion leak conductance [13]. Benzyl- or alkyl- 4-ether-substituted 3-hydroxyproline diastereomers of an (2*S*,3*R*,4*R*) configuration, e.g., **14b** or **14c**, block the anion conductance in both SLC1A4 and SLC1A5 (Figure 3) resulting in an inward current as opposed to the expected outward current induced by substrates with extracellular ^−^SCN buffer (methods). The currents induced by substrates and blocked by hydroxyproline analogs shared a reversal potential shown in the current/voltage relationship consistent with the conductance originating from the same ion pore (Figure 3, inset). A more pronounced current was inhibited in SLC1A5, suggesting a larger anion leak conductance. Particular to AHPCs, each (2*S*, 3*R*, 4*R*)-3-hydroxy-4-o-methyl substituted proline demonstrated a concentration-dependent and saturable block of the leak current (Figure 3C,D). The observed dose–response relationships are independent of competing substrates, and thus provide a direct measure of the equilibrium inhibition constant (K_i_). Each synthetic hydroxyproline analog was functionally assessed for its capacity to inhibit SLC1A4 and SLC1A5 leak currents with affinities reported in Table 1 as determined or approximated K_i_.

#### 2.4.2. BPOHP Pharmacology

**Figure 4:**
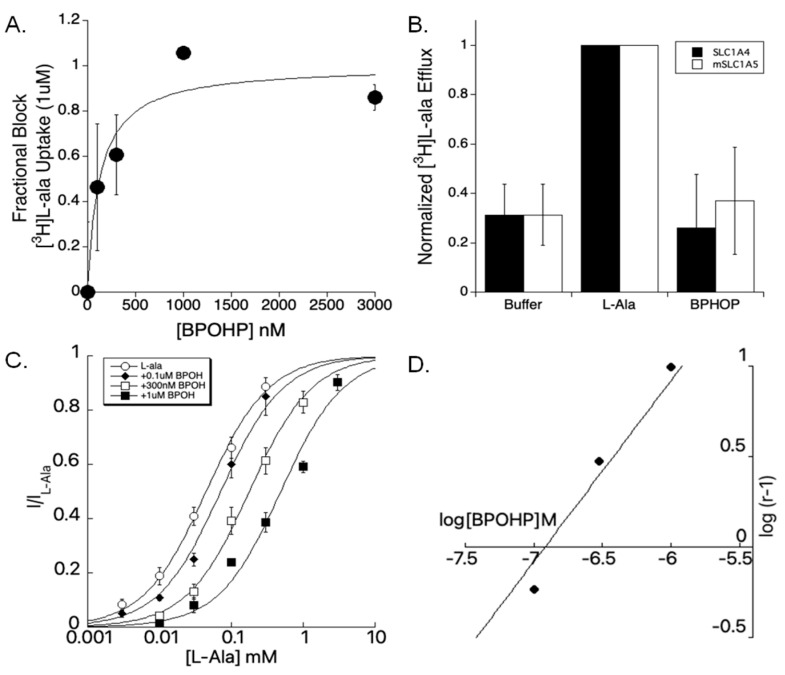
BPOHP **14e** characterization as a competitive, non-transportable inhibitor. (**A**) SLC1A4 injected oocytes where [^3^H]l-alanine uptake is blocked by BPOHP (IC_50_ = 128 nM). (**B**) Radiolabeled [^3^H]l-alanine exchange for SLC1A4 and SLC1A5. Exchange was normalized to the amount induced by 1 mM l-alanine. No exchange was detected in the presence of 3 µM BPOHP. (**C**) l-alanine dose–response curves for SLC1A4, showing the right shift with increasing concentrations of BPHOP (0, 0.1, 0.3, 1 µM). (**D**) A Schild plot showing a linear relationship between [l-ala] dose ratio and four different concentrations of BPOHP, consistent with competitive inhibition.

We further characterized the highest affinity blocker discovered from our AHPC series, biphenyl-O-hydroxy-proline (BPOHP, **14e**). Radiolabeled [^3^H]l-alanine (1 µM) was used to monitor both uptake as well as transporter exchange. BPOHP blocked [^3^H]l-alanine uptake with a slightly lower yet comparable affinity as determined from inhibition of the anion leak current for SLC1A4 (Figure 4A: IC_50_ = 128 nM). To determine whether BPOHP was a substrate or non-transportable blocker, [^3^H]l-alanine was preloaded into oocytes expressing SLC1A4 or SLC1A5 followed by trans-stimulation to demonstrate 1 mM l-alanine, unlike 3 µM BPOHP, induces exchange (Figure 4B). To investigate the nature of BPOHP competitive binding at SLC1A4, we measured l-alanine dose responses in the presence and absence of BPOHP. A respective right shift in the substrate dose response was apparent with increasing concentrations of BPOHP (Figure 4C). A Schild analysis, plotting the log plot of the dose ratio vs a log of inhibitor concentration, revealed a linear relationship, characteristic of competitive inhibition (Figure 4D, K_B_ = 120 nM).

BPOHP activity was evaluated against the glutamate transporters paralogs within the SLC1A gene family (SLC1A 1–3) as well as at neutral amino acid transporters that share common ligand substrates with the SLC1 family including SLC38A1,2, and 4 (ATA1, ATA2, ATA3) and heteromeric transporters SLC7A10+SLC3A2 (asc-1/4f2). Using TEVC, BPOHP did block a 10 µM glutamate current at higher concentrations with the largest overlap with EAAT2 (Figure S2—IC_50_ for EAAT1 = 2.45 µM, EAAT2, 1.2 µM, and EAAT3 = 25.3 µM). Oocytes expressing SLC38A1,2, and 4 transporters produce electrogenic coupled currents in response to l-alanine (100 µM); these currents showed no discernible block at 3 µM BPOHP. Finally, radiolabeled [^3^H]d-serine uptake was used to assess the electroneutral SLC7A10+SLC3A2 transporter, in which 10 µM BPOHP had no effect (Figure S3).

### 2.5. Computational Docking

**Figure 5:**
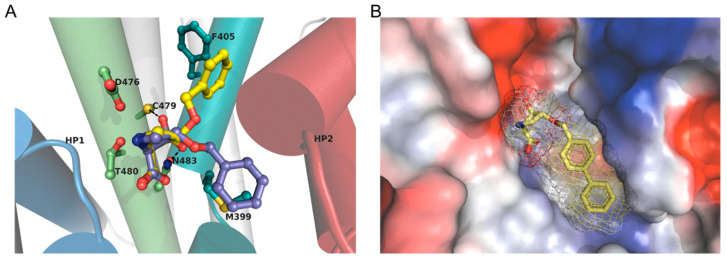
Molecular docking: (**A**) AHPC **17c** (yellow) and **14c** (purple) overlay docked (Autodock 4.2) into an SLC1A5 homology model illustrating two probable hydrophobic pockets, one near phenylalanine F405 and the other on above methionine M399 on TM7 (teal). (**B**) A surface vacuum electrostatics rendering (Pymol) of SLC1A5 depicting high affinity compound **14e** docked into the putative ligand binding site within an outward-facing conformation.

Docking suggests AHPC orientation within the substrate ligand binding pocket/orthosteric site. Synthetically generated AHPCs were compared through computational docking within a SLC1A5 homology model derived from a ligand bound co-crystal structure of Glt_Ph_, 2NWW [53]. The first AHPC with an observable K_i_ for SLC1A4 and SLC1A5 below 100 µM, **14c,** was docked into the SLC1A5 homology model positioning the pyrrolidine amine in contact with the *β*-carboxylic acid of D476 (D464, human equiv.) on transmembrane 8 (TM8, green) and to the nearby T480 (Figure 5A, purple). The *α*-carboxylic acid of **14c** aligned near the primary amide of nearby N483 (human equiv. N471) on TM8 and serine backbone amide proton (S365) of the HP1 loop (blue). Moreover, the (3*R*)-alcohol of AHPC **14c** aligns for a polar interaction with the thiol of C479 (human equivalent C467). Within this binding orientation, the limited conformational freedom afforded by the pyrrolidine ring only permits the benzyl ether moiety of **14c** to position over methionine M399 between the α-helix of transmembrane seven (TM7a; teal) and the HP2 loop (red), a comparable outward-facing conformation observed with the SLC1 family [54]. Conversely, although the benzylic ether orientates within a plausible hydrophobic pocket perpendicular the phenyl ring of F405 (Figure 5A) [55], docking solutions of *cis*-3-hydroxy-*trans*-4-benzyloxy-l-proline **17c** (yellow), an inactive diastereomer, do not depict feasible polar interactions with C479. Like **17c**, docking of diastereomer (2*S*, 3*R*, 4*S*)-3-hydroxy-4-phenoxy proline **12c** into our homology model positions the phenoxy substituent adjacent to the aromatic ring of F405, though unlike **17c** places the 3*R*-alcohol within range to interact with C479, as comparable to **14c**. Taken together, our experimental results, electrophysiologic recordings and computational docking, underscore the contribution to ligand binding afforded by polar interactions with the C479 thiol and/or engagement with the hydrophobic pockets near residue F405 or adjacent to methionine M399.

Computational docking of our highest affinity inhibitor **14e** predicts a hydrophobic pocket that extends from M399 and along TM7α (Figure 5B) and total free energy of binding of (−(8.3–8.7) kcal/mol). The orientation and position of **14e** is consistent with the docking solutions for **14c** where again the (3*R*)-alcohol is in contact with C479, the pyrrolidine amine is positioned with contacts between D476 and T480, and the *α*-carboxylic acid interacting with the primary amide of N483 and serine amide (S365) backbone of HP1. Consistent with the experimentally determined affinities, reported in Table 1, the larger arene substituted AHPCs, **14d**–**f**, are predicted to bind within the ligand binding pocket of SLC1A5 more favorably (lower calculated free energy of binding) than the unsubstituted benzylic-ether of **14c**.

## 3. Methods

### 3.1. Chemicals and Reagents 

[^3^H]l-Alanine and [^3^H]l-glutamine were purchased from Dupont NEN (Boston, MA, USA). *cis*-4-hydroxy-l-proline (**1**), *trans*-4-hydroxy-l-proline (4-HP, **2**), *trans*-3-hydroxy-l-proline (3-HP), and benzyl alcohols were obtained from Acros Organics (Morris Plains, NJ, USA). LC-MS grade acetonitrile for HPLC was obtained from EMD Chemicals USA (Gibbstown, NJ, USA). All other chemical reagents were purchased from Acros Organics (Morris Plains, NJ, USA). All ACS reagent grade EMD brand solvents were purchased from VWR International (Radnor, PA, USA). Separation of regioisomers and isolation of final products were performed on a HPLC C18, 250 mm × 21.2 mm, 10 µm column using a Waters 486 Tunable Detector (Millipore Inc. Milford, MA, USA) and a Milton Roy constaMetric 3000 pump. Full experimental details are reported in the experimental section and Supplementary Materials.

### 3.2. Expression and Functional Testing of Amino Acid Transporters Expressed in Xenopus Laevis Oocytes

The molecular pharmacology of amino acid transport systems SLC1A1–5 (hEAAT3, hEAAT2, hEAAT1, SLC1A4 (hASCT1), SLC1A5 (mASCT2), SLC38A1 (hATA1), SLC38A2 (hATA2), SLC38A4 (hATA3), and SLC7A10+SLC3A2 (hasc-1 + h4f2) were characterized by heterologous expression in *Xenopus laevis* oocytes. Radiolabeled uptake and electrophysiology measurements were made as previously described [10]. Briefly, oocytes were injected with approximately 50 ng of cRNA transcribed from cDNA. For radiolabeled uptake competition experiments, oocytes injected with transporter cRNA were incubated over 10 minutes in ND96 buffer with indicated concentrations of [^3^H]l-alanine or [^3^H]l-glutamine (American Radiolabeled Chemicals; 40–60 Ci mmol^−1^) with or without competing ligands (1 µM). Uptake was halted by washing 3 times with 4 °C ND96 buffer. Oocytes were then lysed in 0.1% Triton X-100, and radioactivity was counted via liquid scintillation. Dose response curves were generated by fitting fractional inhibition (I) to the expression I = I_max_ × [inhibitor]/([inhibitor] + IC_50_). For radiolabeled heteroexchange assays, oocytes were incubated for 20 minutes with radiolabeled 100 µM [^3^H]l-alanine (4 µCi/mL) in frog ringer’s solution. Ligand substrates or non-substrate inhibitors, as indicated, were then assayed for their capacity to induce exchange/release of intracellular radiolabeled [^3^H]l-alanine over 20 minutes by transferring 5 oocytes/well to 500 µL buffer and sampling the buffer for radiolabeled [^3^H]l-alanine every 5 min.

Two-electrode voltage clamp (TEVC) transporter currents were recorded with a GeneClamp 500 amplifier and Digidata 1320A analog–digital interface (Molecular Devices, San Jose, CA, USA). Electrodes (0.2–1.0 MΩ) were filled with 3M KCl and the recording chamber was grounded with an agar bridge. Data were acquired with Axograph (v1.7.6) and LabChart (v7) and analyzed with Kaleidagraph software (v4.1) (Synergy, Reading, PA, USA). Uncoupled anion currents associated with the transport of substrate and block of SLC1A4 or SLC1A5 mediated leak conductance were recorded in the presence of Ringer with 50% substitution of NaCl by NaSCN to amplify the non-transported anion conductance.

### 3.3. Computational Models and Docking 

SLC1A5 or SLC1A4 primary sequence (GenBank accession number D85044 or L14595 respectively), was aligned with the Protein Data Bank (PDB) sequence for the archaeal homologue Glt_Ph_ co-crystallized with non-substrate inhibitor l-*threo*-β-benzyloxyaspartate TBOA, 2NWW.pdb [53]. A SLC1A5 homology model was constructed via sequence alignment along 2NWW coordinates using T-Coffee–Fasta alignment [56], (https://tcoffee.crg.eu/apps/tcoffee/do:expresso (accessed on 1 November 2010) and homology sidechain prediction with SCWRL1.0 (2010)–server (2023, SCWRL4.0, https://bio.tools/scrwl (accessed on 1 November 2010)). The homology model was then optimized through local energy minimizations of regions with high steric and electrostatic interference using the AMBER7 force field in SYBYL (Suite, 2010). Energy minimized (SYBYL) hydroxyprolines were docked into our final homology model using Autodock Tools and Autodock 4.2 [57]. The top ten clusters (poses) from each of three separate docking experiments for each synthesized hydroxyproline were evaluated for their capacity to interact with SLC1A5 residues C479 and D476. The top scoring poses as determined by Autodock 4.2, lowest predicted –ΔG (kcal/mol or kJ/mol) values were assessed and merged into the homology model for visualization in PyMOL Molecular Graphics-System-Version 1.3, SchrödingerLLC (New York, NY, USA).

## 4. Discussion

Here, we demonstrate the beginnings of a structure–activity relationship (SAR) investigation of hydroxyprolines as inhibitors of the neutral amino acid transporters SLC1A4 and SLC1A5. Both transporters exhibit great promise as biologic targets for therapeutic intervention due to their respective roles in d-serine transport within brain tissue [30] and in glutaminolysis in cancerous cells [58]. We discuss, through design and activity-based assessment combined with computational evaluation, the iterative development of a series of AHPCs that led to the discovery of BPOHP, a first-in-class nanomolar inhibitor of SLC1A4 and SLC1A5.

*Trans*-4-hydroxy-l-proline (4-HP), a previously identified substrate for SLC1A5 (ASCT2) [45], induced our discovery of *trans*-3-hydroxy-l-proline (3-HP) as a high affinity substrate of SLC1A4 and SLC1A5. The hydroxyproline diastereomer *cis*-4-hydroxy-l-proline **1** did not induce detectable substrate currents and only blocked the leak anion current above 500 µM. Having observed 3-HP and 4-HP as substrates of SLC1A4 and SLC1A5, we launched a synthetic campaign to generate substituted hydroxyprolines that aid in elucidating crucial molecular interactions and ligand binding mode within the ligand binding site of SLC1A4 or SLC1A5. Among the first hydroxyproline analogs produced in this study, (2*S*,3*R*,4*R*) *trans*-3-hydroxy-*cis*-4-methoxy-l-proline **14a** demonstrated modest inhibitory like activity across SLC1A4 and SLC1A5, K_i_ = 250 µM. Surprisingly, the methyl analog, *trans*-3-hydroxy-*cis*-4-methyl-l-proline **10a**, showed no inhibition of transport, a distinction that may be attributed between the two hydroxyprolines having adopted different conformations, e.g. envelope vs half-boat, and ring pucker. Of importance, (3*S*)-hydroxyprolines **17a**, **17b**, and **17c** were also produced, though demonstrated weak inhibition at SLC1A4 or SLC1A5, K_i_ > 500 µM, and thus, diastereomers of this configuration were not further pursued.

Initial extension of the (4*R*)-methoxy substituent of **14a** to produce the (4*R*)-isopropoxy hydroxyproline **14b** did not improve affinity; therefore, nucleophilic addition using 6-membered arenes were explored. Nucleophilic addition with phenol to eventually produce (3*R*,4*R*)-phenoxy hydroxyproline **12a** provided a measurable increase in affinity. However, a noteworthy increase in affinity for SLC1A4 and SLC1A5 came by extension of methoxy hydroxyproline **14a** to arrive at **14c**, a 4*S*-benzyloxy hydroxyproline. Docking of benzyloxy **14c** and phenoxy **12a** hydroxyprolines scored comparably within our SLC1A5 model, with predicted binding free energy of ~5.5–5.8 kcal/mol; however, **14c** demonstrates a ~6-fold higher affinity for SLC1A4 and SLC1A5 than the phenoxy- hydroxyproline **12a** (Table 1). This difference likely stems from greater conformational flexibility afforded through the methylene-ether connectivity of **14c**, allowing the benzyl-arene to position between the HP2 loop and adjacent hydrophobic residue M399, as observed with ligand TBOA in the Glt_Ph_ model [54].

Previous studies have demonstrated a ~250-fold improvement in affinity for targeting the SLC1 glutamate transporters through extension of the benzyloxy moiety of TBOA [59]. Similarly, replacement of the aryl-amide moiety of γ-aryl-aspartamide inhibitors with aromatic fluorene or biphenyl substituents significantly increased affinity for the glutamate transporters [55,60]. Analogously, at SLC1A4 and SLC1A5, an increase in affinity is also observed via extension of the benzyl-ether moiety of **14c** producing biphenyl-, naphthyl-, or diphenyl-ether containing analogs. Of the three analogs tested, BPOHP (**14e**) demonstrated the highest affinity, inhibiting both SLC1A4 and SLC1A5 transporters. Our docking results suggest a plausible extended hydrophobic pocket between HP2 and TM7 that may accommodate arene-substituted analogues of **14d**–**f** of greater size and diversity, e.g., halogen, methyl, methoxy substituted biphenyls, the structurally rigid aromatic fluorene or numerous heteroarene combinations. Based on our SLC1A5 homology model, our docking results predict that the hydrophobic pocket near F405 (F393 human equivalent) confers high affinity binding. Considering that the hydrophobic pocket adjacent to HP2 over F405 is modestly conserved among the various paralogs of the SLC1 family, ligand designs that engage with this plausible hydrophobic pocket may afford increased affinity and improved selectivity profiles. 

High resolution structural models for SLC1A4 and SLC1A5 have been developed [61,62,63]. These models reinforce our SLC1A5 homology model derived from the archaeal glutamate transporter. Within the SLC1 gene family, the most potent competitive inhibitors assume a similar orientation to TBOA in the original Glt_Ph_ crystal structures as does BPOHP docked into a Glt_Ph_ derived homology model (Figure 5B) [53,54]. Alternative binding pockets for SLC1A5 inhibitors have been proposed that occupy a hydrophobic region between TMD7 and HP2 [9,64], however, like **12c** and **17c** in which computational docking positions their respective arenes within this TMD7/HP2 pocket, these inhibitors exhibit low affinity for SLC1A4 and SLC1A5. While preparing this manuscript, inhibitors of SLC1A5 of a similar design to our alkoxy-hydroxyprolines were reported with ligand-bound high-resolution EM models [43,65]. These reported EM models help validate our docking solutions with respect to mode of binding within our homology model. We emphasize that our discoveries and modeling of these hydroxyproline/AHPC inhibitors targeting SLC1A4 and SLC1A5, as documented in the public domain [66,67], predates these reports and is yet to be properly referenced/cited. The most potent hydroxyproline analog identified here, BPOHP, represents a 7–25-fold improvement in affinity for SLC1A4 and SLC1A5 over ligands previously reported [43,65]. Docking of active AHPC diastereomers of the (3*R*)-alcohol configuration suggests a beneficial polar contact with D476 and/or C479 of SLC1A5 that establishes binding within the substrate/ligand binding pocket. Though BPOHP inhibits the glutamate transporters within the low micromolar range, notably, the other AHPCs of the (3*R*) configuration do not. This nonselective SLC1 inhibition, observed with BPOHP, may be attributed to entropic contributions resulting from the biphenyl moiety occupying an adjacent hydrophobic pocket. Conversely, no inhibition was detected with application of BPOHP among the neutral amino acid transporters from the SLC38 gene family (SLC38A1, SLC38A2, SLC38A4) or at the SLC7A10 (asc-1) +SLC3A2 (4f2) transporter (Figure S3).

Although incomplete, our AHPC series has begun to delineate a SAR for non-substrate inhibitors of the SLC1A4 and SLC1A5 transporters and may provide an entry to designing SLC1 member-selective inhibitors. Among the SLC1 family, l-glutamine is transported exclusively by SLC1A5 [9,68]. Previous studies have demonstrated that the residue position R447 within SLC1A1, which confers substrate specificity for glutamate [68] within the SLC1 transporter family, also distinguishes glutamine as a substrate of SLC1A5 in contrast to SLC1A4 [9]. It is worth noting a second mutation in TMD8 was needed to confer l-Gln transport within SLC1A4 which the authors hypothesize is a size constraint of the binding pockets. One possible option in pursuit of SLC1A4 inhibitors might include analogs of *cis*-3-phenyl-4-*trans*-hydroxy-l-proline, **10d**. In this regard, we observed that 3-*cis*-methyl hydroxyproline **10b** demonstrated modest selectivity for SLC1A4 over SLC1A5, which seems to further improve with an arene substituent as observed with **10d**, resulting in an increased affinity for SLC1A4 and no detectable change in affinity for SLC1A5. Also noteworthy, the docking of our most active AHPCs, e.g. BPOHP, suggests a polar interaction between the (3*R*)-alcohol with C479 of SLC1A5 (C467 in humans). Strategies that modify the pyrrolidine scaffold of BPOHP to include thiol specific covalent reactive functional groups might greatly improve inhibition of SLC1A5 particularly. Collectively, these observations will inform future ligand design strategies to discover SLC1 member selective inhibitors. 

Small-molecule inhibitors of SLC1A5, derived from amino acid substrates, have been previously identified, including examples that were initially mischaracterized as SLC1A5 inhibitors, such as V-9302 [69]. Moreover, there are purported SLC1A5 agents that demonstrate broad inhibition across SLC family transporters and/or other off-target effects, which may obfuscate interpretation regarding how SLC1A4 or SLC1A5 contribute to pathophysiologic processes [69,70,71,72,73,74]. Such example reports accentuate the importance of full pharmacologic characterization of small-molecule inhibitors across the SLC glutamine transporters. Although, the highest affinity blocker we identified, BPOHP, marks an advancement in agents targeting SLC1A4 and SLC1A5 which will inform future design of SLC1 family modulators. ‘Lead’ discovery based on AHPCs will necessitate improvements in drug-like properties and SLC1 member selectivity before progressing to preclinical or animal model studies. Continued efforts to develop SLC1A4 and SLC1A5 lead-like inhibitors will focus on optimizing selectivity through substitution of the BPOHP arenes moieties or alternative strategies that explore polar interactions with SLC1A5 residues D476 and/or C479. Besides SLC1 family transporters, other SLC transporters will be considered for activity-based evaluation, such as the neutral amino acid transporters from the SLC38A, SLC7A, and SLC6A gene families which may share similar or overlapping pharmacophores [75].

## Figures and Tables

**Table 1:** Hydroxyproline K_i_ values for SLC1A4 and mSLC1A5. Values were calculated from the dose–response of blocked anion conductance. Compounds tested only once are shown without standard error.

Compound No.	Structure	SLC1A4 K_i_ (µM)	SLC1A5 K_i_ (µM)	Compound No.	Structure	SLC1A4 K_i_ (µM)	SLC1A5 K_i_ (µM)
**1**	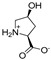	805 ± 148	872 ± 201	**14a**	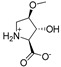	214 ± 32	235 ± 69
**26**	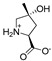	No Block	926 ± 252	**14b**	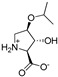	237 ± 40	268 ± 70
**27**	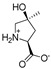	No Block	1077 ± 322	**14c**	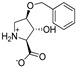	39 ± 5	69 ± 7
**10d**	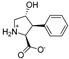	332 ± 74	1128 ± 318	**17a**	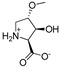	481 ± 77	600 ± 176
**10c**	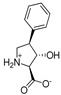	~600	~600	**17b**	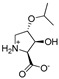	505 ± 105	~400
**10b**	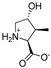	564 ± 149	922 ± 316	**17c**	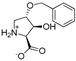	493 ± 61	828 ± 248
**10a**	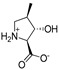	1050 ± 110	1149 ± 283	**14d**	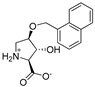	~10	~10
**12a**	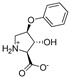	150 ± 43	141 ± 20	**14e**	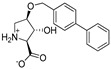	0.31 ± 0.04	0.24 ± 0.04
**12b**	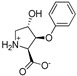	594 ± 191	502 ± 110	**14f**	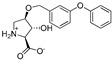	13 ± 2	10 ± 2
**12c**	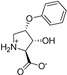	~150	~150				

## Data Availability

Additional information concerning the computational modeling can be obtained from the corresponding author.

## Supplementary Materials

The following supporting information can be downloaded at https://www.mdpi.com/article/10.3390/molecules29102330/s1: full experimental details and characterization of new compounds; spectra for all new compounds from Dr. Lyda’s Ph.D. dissertation are publicly accessible on the University of Montana Scholar Works page: https://scholarworks.umt.edu/cgi/viewcontent.cgi?article=1961&context=etd (accessed on 13 May 2024).

## Abbreviations

AHPC	Alkoxy hydroxy-pyrrolidine carboxylic acids
3-HP	*Trans*-3-hydroxy-l-proline
4-HP	*Trans*-4-hydroxy-l-proline
ASC	Ala, Ser, Cys-selective transporter
BPOHP	Biphenyl-O-hydroxy-proline, structure **14**
gCOSY	Gradient correlation spectroscopy
mCPBA	*meta*-chloroperoxybenzoic acid
NMDA	*N*-methyl d-aspartate
PDB	Protein Data Bank
SAR	Structure–activity relationship
SLC	Solute carrier superfamily
TBOA	l-*threo*-β-benzyloxyaspartate
TEVC	Two-electrode voltage clamp
TM	Transmembrane helix
